# Overexpression of IFN-induced protein with tetratricopeptide repeats 3 (IFIT3) in pancreatic cancer: cellular “pseudoinflammation” contributing to an aggressive phenotype

**DOI:** 10.18632/oncotarget.2494

**Published:** 2014-09-17

**Authors:** Hanno Niess, Peter Camaj, Ruth Mair, Andrea Renner, Yue Zhao, Carsten Jäckel, Peter J. Nelson, Karl-Walter Jauch, Christiane J. Bruns

**Affiliations:** ^1^ Department of Surgery, Medical Center of the Ludwig-Maximilians-University, Campus Grosshadern, Munich, Germany; ^2^ Department of Surgery, Medical Center of the Otto-von-Guericke-University, Magdeburg, Germany; ^3^ Medizinische Klinik und Poliklinik IV, Campus Innenstadt, Klinikum der Universitaet Muenchen, Arbeitsgruppe Klinische Biochemie, Munich, Germany

**Keywords:** IFIT, IFIT3, RIG-G, ISG60, SOX9, inflammation, pancreatic cancer, chemotherapy resistance, cytokine expression

## Abstract

Inflammation contributes to important traits that cancer cells acquire during malignant progression. Gene array data recently identified upregulation of interferon-induced protein with tetratricopeptide repeats 3 (IFIT3) in aggressive pancreatic cancer cells. IFIT3 belongs to the group of interferon stimulated genes (ISG), can be induced by several cellular stress stimuli and by its tetratricopeptide repeats interacts with a multitude of cellular proteins.

Upregulation of IFIT3 was confirmed in the aggressive pancreatic cancer cell line L3.6pl compared with its less aggressive cell line of origin, COLO357FG. Transgenic induction of IFIT3 expression in COLO357FG resulted in greater mass of orthotopic tumors and higher prevalence of metastases. Several important traits that mediate malignancy were altered by IFIT3: increased VEGF and IL-6 secretion, chemoresistance and decreased starvation-induced apoptosis. IFIT3 showed binding to JNK and STAT1, the latter being an important inducer of IFIT3 expression. Despite still being alterable by “classical” IFN or NFκB signaling, our findings indicate constitutive - possibly auto-regulated - upregulation of IFIT3 in L3.6pl without presence of an adequate inflammatory stimulus. The transcription factor SOX9, which is linked to regulation of hypoxia-related genes, was identified as a key mediator of upregulation of the oncogene IFIT3 and thereby sustaining a “pseudoinflammatory” cellular condition.

## INTRODUCTION

Advanced pancreatic cancer is characterized by a highly aggressive phenotype showing infiltrative, fast and destructive growth, with early formation of metastases. The malignant properties of tumor growth are mediated by the cellular accumulation of genetic alterations, which eventually allow for uncontrolled growth of these cells. By loss of tumor suppressor genes, and gain of oncogenes, pre-malignant lesions develop into highly aggressive cancers. However, despite their clonal origin, tumors consist of a heterogeneous mass of cells with different amounts of genetic changes and thus different malignant potential [1]. Hence, only a fraction of tumor cells are capable of invasive growth and formation of metastases.

Inflammation is regarded as a hallmark of cancer and clear links to genetic instability caused by inflammatory processes have been described [2]. Inflammation and cancer are thought to be connected by two pathways: an intrinsic pathway, where genetic alterations in cancer cells result in inflammation [3], and an extrinsic pathway, where inflammatory conditions increase the risk for cancer. Mediators of the inflammatory response such as reactive oxygen and nitrogen intermediates can cause direct genetic alterations but also indirect effects such as inflammation-associated activation of NFκB are important aspects of the tumor-promoting effects of inflammation [4].

The importance of inflammation for tumor-progression can be observed in patients with inflammatory bowel disease, which are at increased risk for the development of colorectal cancer and the use of anti-inflammatory drugs reduces this risk [5, 6]. In addition, chronic pancreatitis is an established risk factor for the development of pancreatic cancer [7] and the profound reactive tumor stroma of pancreatic cancer facilitates a constant pro-inflammatory micromilieu, thereby enhancing tumor growth [8].

Repeated orthotopical injections of COLO357FG pancreatic cancer cells into the pancreas of mice followed by isolation of tumor cells from liver metastases led to the generation of the cell line L3.6pl subtype [9]. L3.6pl is now a well-established cell line, which produces profoundly aggressive tumors in orthotopic animal models. We recently identified differentially expressed genes between the two related cell lines COLO357FG and L3.6pl [10]. Among others, genes associated with inflammation such as interferon receptor 1 (IFNAR1), its down-stream proteins Signal Transducers and Activators of Transcription 1 (STAT1) and IFN-induced protein with tetratricopeptide repeats 3 (IFIT3) were significantly increased in L3.6pl.

The biology of the IFIT family of proteins is complex and far from fully understood. In humans, four family members have been described and partially characterized: IFIT1 (also known as IFN stimulated gene (ISG) 56), IFIT2 (ISG54), IFIT3 (ISG60) and IFIT5 (ISG58) [11]. While expression levels of IFIT genes are low in most tissues, their transcription is strongly induced in many cell types after IFNAR signaling or viral infection [12]. The IFIT genes can also show cell and tissue type specific expression kinetics, which has been proposed to confer non-redundant antiviral actions against specific viral infections [13]. The strongest inducers of IFIT genes identified to date include IFN-α/β, whereas IFN-γ shows weak induction [14].

Signals generated after the ligation of pattern recognition receptors by non-self pathogen-associated molecular patterns (PAMPs; such as double-stranded RNA and lipopolysaccharide) can trigger IFIT gene expression independently of type I IFNs. Following viral infection, IFIT induction is conferred directly by activation of IFN-regulatory factor (IRF) 3, which is often activated before the induction of type I IFN [15]. Other known IRF proteins (such as IRF1, IRF5, IRF7, and IRF9) also have the capability to induce the expression of IFIT genes directly [16, 17]. Furthermore, retinoic acid signaling – although in a slower manner than PAMPs - is also capable of inducing IFIT gene expression [18].

IFIT proteins apparently lack enzymatic activity but share the commonality of containing tetratricopeptide repeats (TPR) motifs spread throughout their sequences. The 34 amino acid long TPR motif adopts a helix-turn-helix structure by which it is involved in protein-protein interactions and the assembly of large protein complexes [19]. Through their interaction capacities with cellular proteins containing TPR motifs themselves, the possible effects of IFIT proteins on cellular functions range from the regulation of gene transcription, cell cycle control, mitochondrial and peroxisomal protein transport, protein kinase inhibition, NADPH oxidase activity, protein folding, immunity and viral replication [20]. Numerous proteins that are capable of binding RNA or that are suspected to be involved in mRNA translation were found to bind to IFIT proteins [21]. But due to the multitude of possible protein-protein interactions of the heteromers formed by IFIT proteins the full repertoire of their functions are still obscure.

IFIT3 was originally identified in promyelocytic leukemia cells stimulated with retinoic acid [18, 22]. It is, like the other IFITs, also upregulated upon cellular stimulation with IFN [23]. In the present study, we investigate the influence of IFIT3 on the malignant potential of pancreatic cancer cells, describe protein-protein interactions involving IFIT3 and elucidate possible mechanisms by which constitutive upregulation of IFIT3 is mediated in L3.6pl.

## RESULTS

### IFIT-3 is over-expressed in the highly aggressive pancreatic cancer cell line L3.6pl

**Figure 1:**
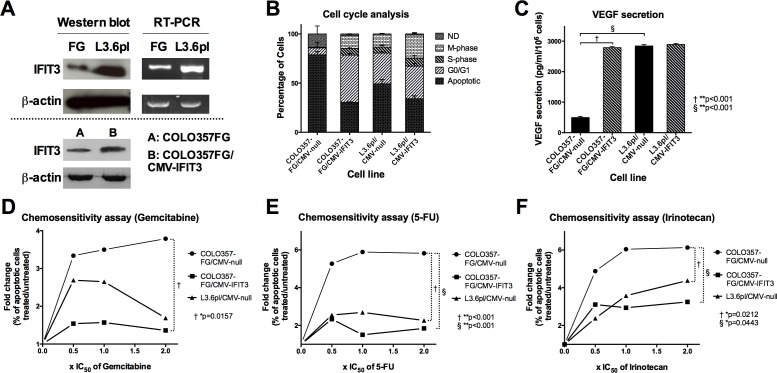
Differential expression of IFIT3 in COLO357FG and L3.6pl and the resulting biological effects (A) Western blot and semi-quantitative RT-PCR of COLO357FG and L3.6pl shows elevated IFIT3 expression in L3.6pl on mRNA and protein level thus confirming gene array data. Bottom: confirmation of stable transfection of COLO357FG with IFIT3 expressing vector (COLO357FG/CMV-IFIT3) by Western blot. (B) Cell cycle analyses of IFIT3-overexpressing and empty vector control cell lines of COLO357FG and L3.6pl (ND=not definable; bars represent mean of triplicate experiment, error bars depict SD). Statistical significance was tested using unpaired t-test for each respective cell phase with the results described in the results section. (C) ELISA of cell supernatant from transfected cell lines assessing concentration of vascular endothelial growth factor (VEGF). Each bar represent mean of triplicate experiment, error bars depict SD. Unpaired t-tests were performed to assess for statistical significance of the differences. (D-F) Chemosensitivity assays illustrating fold-change of percentage of apoptotic cells under increasing concentrations of chemotherapy with gemcitabine (D), 5-FU (E), and irinotecan (F), plotted as multiples of the respective IC_50_ as published elsewhere [47]. Circles represent COLO357FG/CMV-null cells, squares COLO357FG/CMV-IFIT3, and triangles L3.6pl/CMV-null in each graph. Statistical significance was determined using one-way ANOVA analysis with Tukey's HSD post-hoc test.

The metastatic pancreatic cancer cell line L3.6pl, which is a highly aggressive offspring of COLO357FG has previously been evaluated for differential gene expression profiles by gene array [9, 10]. Here, an increase of the steady state level of mRNA for the *IFIT3* gene by 3-fold was seen in L3.6pl cells (database found at www.ncbi.nlm.nih.gov/projects/geo/index.cgi under the accession number GSE9350). To validate this, semi-quantitative RT-PCR and Western blot were performed showing increased expression of *IFIT3* mRNA and protein in L3.6pl (Figure 1A).

### IFIT3 is expressed in human pancreatic cancer samples

To assess expression levels of IFIT3 in human pancreatic cancer samples, gene array data sets published by van den Broeck et al. were analyzed for expression profiles of IFIT3 [24]. The authors collected tumor samples from pancreatic cancer patients that were stratified by good (overall and disease-free survival > 50 months) and poor outcome (disease-free survival < 7 months and overall survival < 19 months) as well as from liver- and peritoneal metastases and performed expression profiling by gene array. The trend showed that the expression of IFIT3 was found to be increased (1.53 times higher) in pancreatic cancer samples of patients with poor outcome as compared to patients with a good outcome (mean expression levels of 697.14 (±473.22) vs. 456.11 (±103.25), q-value=0.530, regularized t-test, 21 probes in the set). In the analysis of probes obtained from metastases vs. those of tumors from patients with good outcome, a 1.4-fold higher expression level of IFIT3 (555.72 (±311.36) vs. 396.97 (±86.28), q-value=0.368, regularized t-test, 21 probes in set) was observed. Comparison of the expression levels of the housekeeping genes ubiquitin B (UBB), cadherin 4, type 1, R-cadherin (retinal) (CDH4), glyceraldehyde-3-phosphate dehydrogenase (GAPDH), and prefoldin subunit 1 (PFDN1) showed non-significant fold-changes in the comparison between good and poor outcome of −1.07, −1.09, −1.04, and 1.08, respectively, and fold-changes in the comparison between metastases and good outcome of 1.00, 1.05, 1.01, and 1.07, respectively.

### Transgenic expression of IFIT3 in COLO357FG and L3.6pl rescues cells from starvation-induced apoptosis, increases proliferation rate, resistance to chemotherapy, and VEGF secretion

In order to assess the biological influence of IFIT3 expression on the malignant potential of pancreatic cancer cells, COLO357FG cells and L3.6pl cells were stably transfected with either vectors resulting in over-expression of IFIT3 (CMV-IFIT3) or empty vectors as control (CMV-null) (figure 1A, bottom). IFIT3 expression was driven by the constitutively active CMV promoter and thus was independent of physiological regulatory processes for IFIT3 expression.

Cell cycle analysis following serum deprivation (figure 1B) revealed that transgenic overexpression of IFIT3 in COLO357FG/CMV-IFIT3 led to a reduced percentage of apoptotic and a higher proportion of proliferating cells in the S- and M-phase as compared to COLO357FG/CMV-null cells [proportion of apoptotic cells: 78.8 (±3.6)% vs. 30.3 (±0.7)%, **p<0.001; S-phase: 0.2 (±0.1)% vs. 6.7 (±1.5)%, *p<0.01; M-phase: .4 (±0.2)% vs. 13.3 (±0.6)%, **p<0.001]. Transgenic upregulation of IFIT3 expression in the already IFIT3-high expressing L3.6pl (L3.6pl/CMV-IFIT3) had a less profound effect on the proportions of apoptotic and proliferating cells [L3.6pl/CMV-null vs. L3.6pl/CMV-IFIT3: apoptotic cells: 49.2 (±4.4)% vs. 34.1 (±3.0)%, *p<0.01; S-phase: 6.0 (±2.0)% vs. 8.0 (±3.0)%, not significant; M-phase: 13.0 (±1.0)% vs. 23.7 (±2.5)%, *p<0.01].

Increased secretion of the vascular endothelial growth factor (VEGF) is linked to more aggressive tumor growth in pancreatic cancer. VEGF ELISA was performed on culture supernatants from control cell lines and IFIT3 overexpressing cell lines. Overexpression of IFIT3 resulted in a 5.6-fold increase in VEGF secretion (**p<0.001). The VEGF concentration in the culture medium did not differ significantly to that seen in L3.6pl/CMV-null. Transfection of L3.6pl with an IFIT3 overexpressing vector did not increase VEGF secretion in L3.6pl cells.

Resistance to chemotherapeutic drugs represents an important attribute of “aggressive” tumors. COLO357FG/CMV-null showed a rapid rise in the proportion of apoptotic cells when treated with increasing concentrations of the chemotherapeutic agents gemcitabine, 5-FU, and irinotecan (figure 1D/E/F; circles). Overexpression of IFIT3 (squares) caused a significant gain of resistance towards all chemotherapeutic agents assessed. L3.6pl/CMV-null (triangles) also proved significantly more resistant to chemotherapy than COLO357FG cells in case of 5-FU and irinotecan. No significant difference was observed between COLO357FG/CMV-IFIT3 and L3.6pl/CMV-null.

### Transgenic upregulation of IFIT3 in COLO357FG cells results in enhanced orthotopic tumor growth and formation of more metastases *in vivo*

To assess the influence of IFIT3 expression on tumor formation capabilities and growth characteristics *in vivo*, the transgenic pancreatic cancer cell lines were orthotopically injected into Balb/c nu/nu mice. COLO357FG/CMV-IFIT3 cells showed higher rates of tumor engraftment and a greater median tumor mass than COLO357FG/CMV-null. However, tumors that formed after injection of L3.6pl resulted in even heavier tumors and a similar tumor incidence to that seen after COLO357FG/CMV-IFIT3 inoculation.

**Figure 2:**
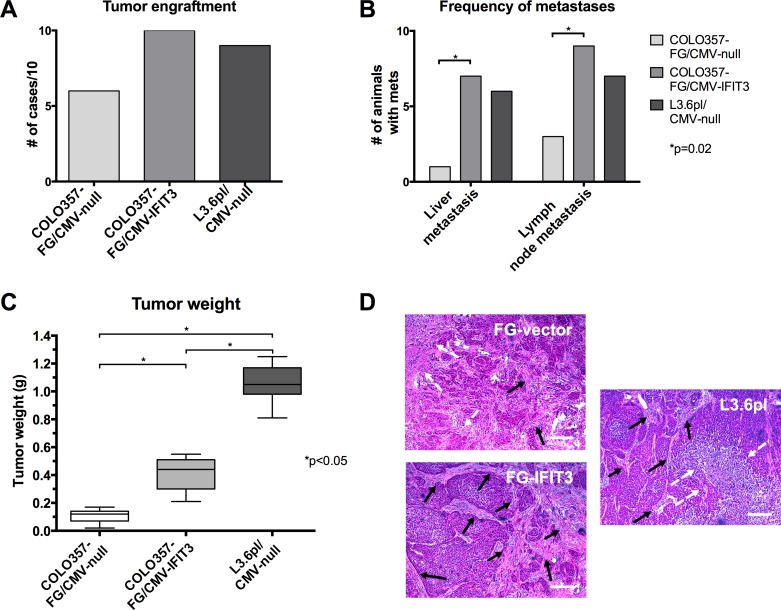
Effects of IFIT3 expression on orthotopic tumor formation and growth Orthotopic injection of tumor cell lines was performed into the pancreas of nu/nu mice (n=10 for each group). (A) The graph depicts the number of successful tumor engraftments leading to visible tumors in the pancreas after sacrifice of the animal. (B) Graph shows the number of respective animals that developed liver or lymph node metastasis, which were defined to be visible by eye and exceed 1 mm in diameter. Fisher's exact tests were used to determine statistical significance. (C) Box plots with the line representing median, whiskers representing range and boxes representing interquartile range of tumor mass for each group. Statistical significance between groups was tested using Mann-Whitney-U tests. (D) Representative H&E stains of paraffin-embedded tumor slides. A more profound desmoplastic reaction was observed in IFIT3 overexpressing tumors (black arrows: stromal tissue, white arrow: necrosis, bars: 200 μm).

Tumors derived from COLO357FG/CMV-IFIT3 cells showed a significantly higher prevalence of liver- and lymph node metastases at the time of sacrifice as compared to empty vector control cells, while not differing significantly from L3.6pl/CMV-null derived tumors (figure 2B).

Pancreatic tumors were isolated after sacrifice of the animals and examined by microscopy. H&E staining of tumor sections revealed a more profound desmoplastic reaction with larger areas of connective tissue in tumors from mice that received the IFIT3 overexpressing variant of COLO357FG cells as compared to the empty vector controls (figure 2D).

### IFIT3 interacts with JNK and STAT1

**Figure 3:**
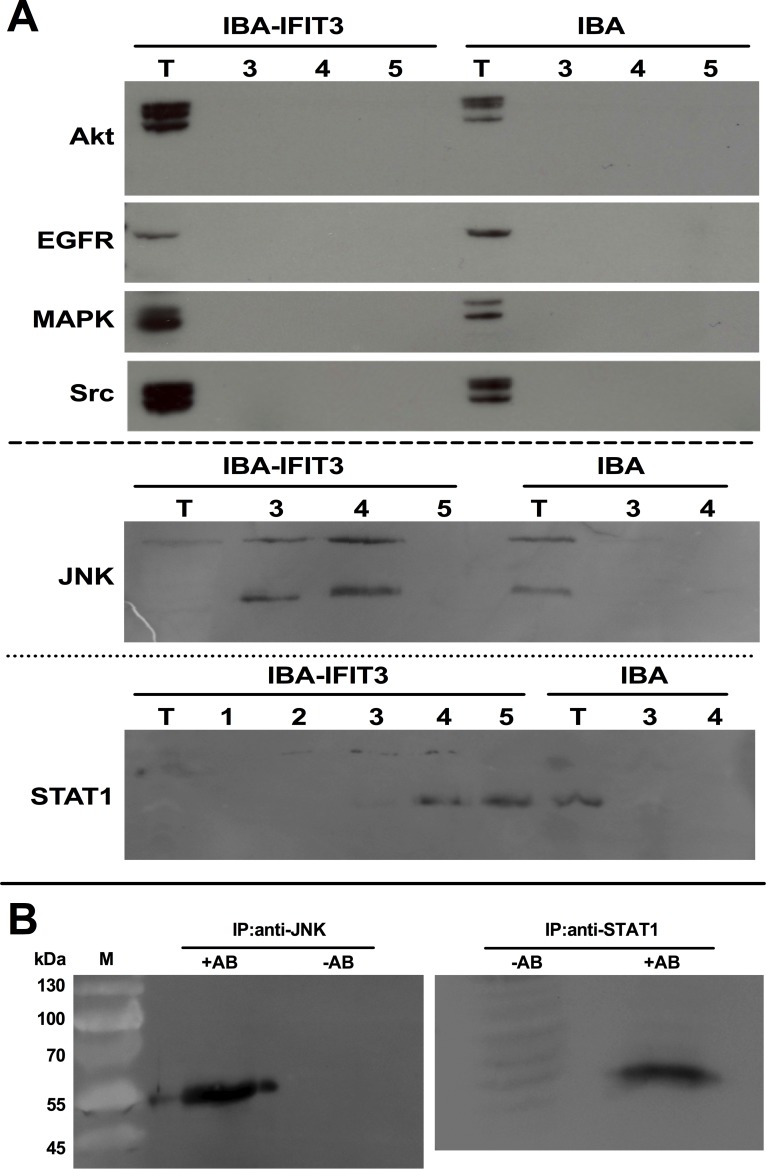
Protein:protein interactions of IFIT3 Western blots following. (A) Following protein affinity chromatography using an IFIT3-STrEP-tag® fusion protein in L3.6pl, possible binding partners of the IFIT3-STrEP-tag® were explored by Western blots using specific antibodies. Anti-Akt, anti-EGFR, anti-MAPK and anti-Src antibodies were not able to detect the IFIT3-STrEP-tag® fusion protein. In the anti-JNK and anti-STAT1 Western blots, positive bands were visible (T=total cell extract, 1-5: fractions). (B) The positive bands were confirmed by performing immunoprecipitation using anti-JNK and anti-STAT1 antibodies followed by Western blots with an anti-IFIT3 antibody.

IFIT3, like the other members of the IFIT-family, contains unique helix-turn-helix motifs termed tetratricopeptide repeats (TPRs), which may mediate a variety of protein–protein interactions [20]. Using the One-STrEP purification method, possible protein-protein interactions involving IFIT3 were examined. Akt, EGFR, Src, MAPK, STAT1 and JNK, all key proteins of pathways involved in cell proliferation, survival, inflammation, etc. were assessed as possible candidates. After labeling IFIT3 with the STrEP Tag, protein complexes that formed after interaction of IFIT3-StrEP with proteins from the tumor cell lysate stained positive for JNK and STAT1 by Western Blot analysis, while other typical signaling proteins such as Akt, EGFR, MAPK and Src were not detectable (figure 3A). Reciprocally, using anti-JNK and anti-STAT1 antibodies allowed for isolation of protein complexes that contained IFIT3 as shown by positive anti-IFIT3 Western blot results (figure 3B).

### IFIT3 expression in both FG and L3.6pl pancreatic cancer cell lines is stimulated by IFN-alpha and dependent on NF-κB and STAT1 signaling

IFIT3 belongs to the group of interferon-stimulated genes (ISGs) and induction of IFIT3 in response to IFN-α stimulation has been described in several cell lines, suggesting a role for this protein in IFN-α-mediated actions [25]. The Janus activated kinase/signal transducer and activator of transcription (JAK/STAT) pathway mediates ISG expression following interferon signaling [26]. Although alternate routes of IFIT3 induction, independent from STAT1 are known, STAT1 appears to be a key factor for enhanced IFIT3 expression [27]. The nuclear factor κB (NFκB) protein c-Rel has been shown to act as a regulator for the expression of several ISGs, including IFIT3 [28].

**Figure 4:**
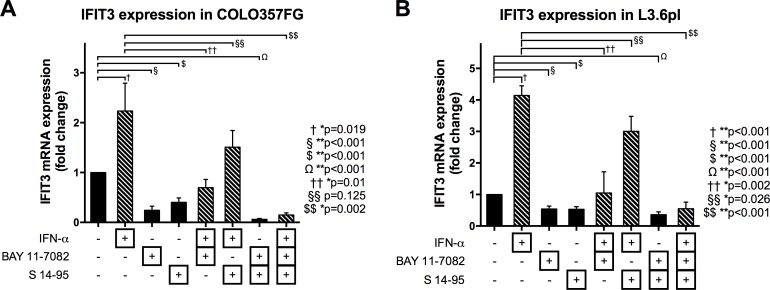
Expression changes of IFIT3 mRNA depending on stimulation with IFN and blockade of NFκB and STAT1 signaling Quantitative PCRs were performed on COLO357FG (A) and L3.6pl (B) cells. Bars represent means of triplicate experiments with error bars representing SD. Expression changes are shown as fold-changes as compared to unstimulated cells (bar #1 in each graph). Cells were either treated with IFN-α, BAY 11-7082 (to block NFκB signaling), or S 14-95 (to block STAT1 signaling) or any combination of the three. Statistical analysis was performed using unpaired t-tests.

In both COLO357FG and L3.6pl cells, steady state expression of IFIT3 was significantly reduced by treatment with specific inhibitors for NFκB (BAY 11-7082) or STAT1 (S14-95) (figure 4).

IFN-α stimulation on the other hand resulted in significant induction of IFIT3 mRNA expression. Although the stimulatory effects of IFN-α on IFIT3 expression was reversible by inhibition of NFκB signaling in both COLO3567FG and L3.6pl cells and by STAT1 inhibition in L3.6pl cells, the expression levels were still equal or even higher than that seen in unstimulated cells. In the COLO357FG cells, even after blockade of both NFκB and STAT1 signaling, the stimulation of cells with IFN-α led to a significant increase in IFIT3 expression levels.

### SOX9 positively regulates IFIT3 expression in the pancreatic cancer cell line L3.6pl

**Figure 5:**
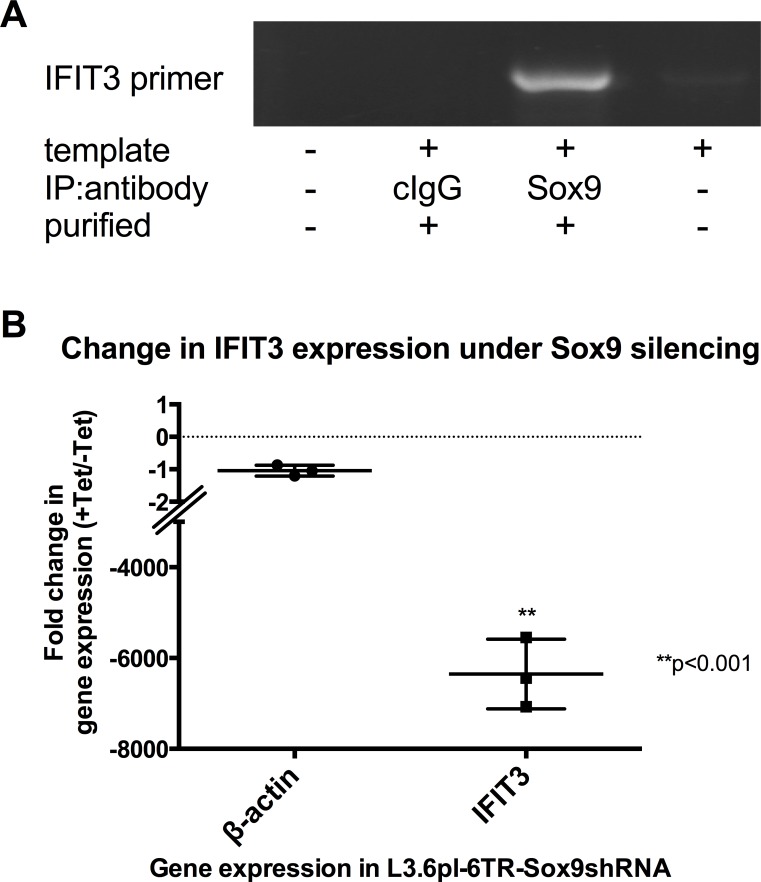
Dependency of IFIT3 expression on SOX9-mediated signaling (A) Chromatin immunoprecipitation (ChIP) against SOX9 in L3.6pl cell lysate followed by PCR using specific primers for detection of the IFIT-3 primer (PCR product identity confirmed by sequencing) demonstrates covalent binding of SOX9 on the IFIT3 primer. No template, no antibody and unspecific IgG antibody for immunoprecipitation were run as negative controls. (B) The graph depicts results from quantitative PCRs of IFIT3 mRNA expression in L3.6pl-6TR-SOX9shRNA. Addition of tetracycline to these transfected cells induces the expression of SOX9shRNA, which efficiently silences SOX9 expression (confirmed by Western blot in [29]). The plots represent results from triplicate experiments and are presented as fold-changes in the cell line with or without addition of tetracycline. Expression level changes of IFIT3 were compared to expression changes of the housekeeping gene β-actin as control. Significance was tested using an unpaired t-test.

The transcription factor SOX9 was recently shown to be increased in L3.6pl independently of the presence of hypoxic or normoxic conditions and to have pancreatic tumor-promoting effects [29]. Bioinformatics analysis of the immediate upstream promoter region of the IFIT3 gene using Lasergene® GeneQuest™ software identified a potential SOX9 binding site. To test for direct binding of SOX9 at his site, chromatin immunoprecipitation (ChIP) was performed on cell lysates from L3.6pl cells using an anti-SOX9 antibody. The chromatin-protein complexes retrieved by this assay showed the presence of the IFIT3-SOX9 binding region using IFIT3 primers (figure 5A).

Transfection of L3.6pl with a gene vector that allows for tetracycline-inducible expression of SOX9shRNA (L3.6pl-6TR-SOX9shRNA) was used to generate a functional knockdown of SOX9 expression in L3.6pl cells. Silencing of SOX9 by this construct was demonstrated in [29]. Silencing of SOX9 led to a parallel down-regulation of IFIT3 mRNA expression [6350 (±443)-fold]. SOX9 is thus responsible in part for the constitutive upregulation of IFIT3 expression seen in L3.6pl, which is independent from hypoxic conditions or external signaling, e.g. IFN signaling (figure 5B).

### IFIT3 induces Il-6 expression independently of IFN signaling, silencing of SOX9 inhibits this induction

To support the hypothesis that IFIT3 expression is factually accompanied by an “inflammatory status” in pancreatic cancer cells, we examined the secretion of the inflammatory cytokines IL-6, TNF-α, IL-1ß, IL-2, and IL-10 with respect to the expression of IFIT3 and SOX9 in respective culture supernatants.

**Figure 6:**
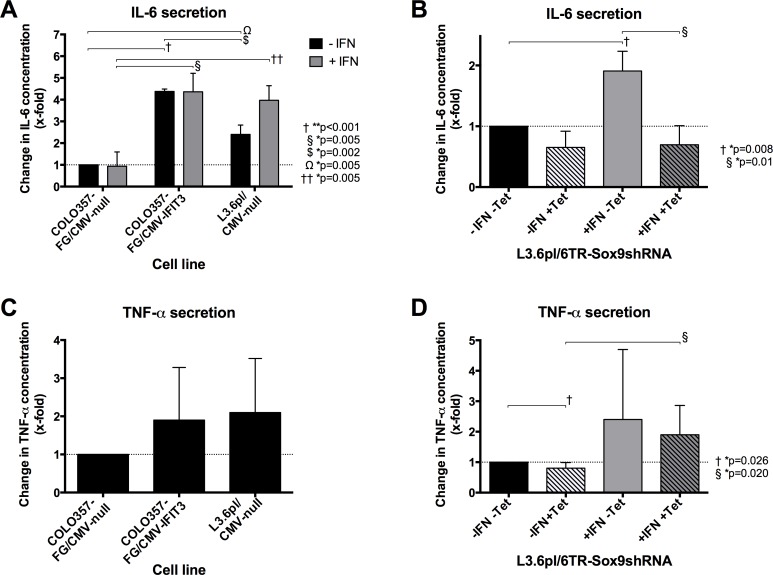
Effects of IFIT3 and SOX9 overexpression on secretion levels of the inflammatory cytokines Il-6 and TNF-α (A) ELISA assessing the concentrations of Il-6 in supernatants of COLO357FG/CMV-null, COLO357/CMV-IFIT3 and L3.6/CMV-null without (black bars) or with (grey bars) previous IFN-α treatment. (C) The graph shows results of ELISAs on the same cell lines assessing TNF-α concentrations but without IFN treatment. (B&D) ELISAs of IL-6 (B) and TNF-α (D) concentrations were performed on the supernatant of L3.6pl-6TR-SOX9shRNA. Tetracycline can be used to induce silencing of SOX9 by SOX9shRNA (striped bars). IL-6 concentration was assessed also depending on IFN-α treatment of the cells (grey bars). Bars in all graphs represent mean values of triplicate experiments with error bars representing the SD. All values are shown as fold-changes compared to the respective concentrations measured in untreated COLO357FG/CMV-null (A/C) and L3.6pl-6TR-SOX9shRNA cells (B/D), respectively (bars #1 in each graph). Statistical significance was tested using unpaired t-tests.

In cells that were not stimulated with IFN-α (figure 6, black bars), IL-6 protein was significantly increased in response to IFIT3 overexpression. Stimulation of the cells with IFN-α (figure 6A, grey bars) showed a similar picture of IL-6 protein in the cell supernatant. While no increase in IL-6 protein was seen in the COLO357FG cells, levels were dramatically increased in the L3.6pl cells after IFN-α treatment.

Silencing of SOX9 by tetracycline-inducible expression of SOX9shRNA in L3.6pl (figure 6B, striped bars) resulted in a small and statistically insignificant decrease of IL-6 concentration in cells that were not stimulated with IFN-α (black), but in a significant reduction of IL-6 protein in IFN-stimulated cells (grey bars) as compared to the respective cell lines in which SOX9 silencing by tetracycline treatment was not performed (solid bars).

Experiments were run with L3.6pl-6TR-LacZshRNA cells as controls to rule out inflammatory stimuli by the shRNA vector itself. No IL-6 induction with tetracycline treatment was seen (data not shown).

Although the TNF-α concentration was higher in IFIT3 overexpressing cells, they did not differ significantly (figure 6C). Silencing of SOX9 in L3.6pl (figure 6D, striped bars) resulted in a significant reduction of the TNF-α concentration in cells that were not stimulated with IFN-α (black bars). On the other hand, silencing of SOX9 could not abrogate the increase of TNF-α secretion in L3.6pl following IFN-α stimulation (grey bars).

The cytokines IL-1β, IL-2, and IL-10 were not detectable in the supernatants of the cells at a concentration that allowed for comparison (data not shown).

## DISCUSSION

We show that the increased expression of IFIT3 is in part responsible for the more aggressive phenotype seen in the pancreatic cancer cell line L3.6pl as compared to its parental line COLO357FG [10].

IFIT proteins are expressed by cells upon stimulation with IFN, all-trans retinoic acid, viral infection, exposure to dsRNA, or lipopolysaccharide (LPS) and are thought to play a role in the response to cellular stress [30]. Despite the similarities of in their primary structures, the biological functions of the IFIT proteins are distinct from another and there is cell type-specific and inducer-specific differential induction of the IFIT genes [19]. IFIT3 is capable of forming heteromers with other proteins from the IFIT family, and with other proteins from the cytoplasm that contain TPR motifs.

The Human Protein Atlas project (www.proteinatlas.org) encompasses data on protein expression profiles encoding for over 16.000 genes in 20 different cancer types. Analysis of this database showed that IFIT3 was detectable at the protein level by tissue microarray in 5 out of 11 pancreatic cancer samples while no expression was seen in normal pancreas. In addition, IFIT1 was detectable in normal pancreas and in 9 out of 11 cancer samples, while IFIT2 and IFIT5 could not be found in either normal pancreas or pancreatic cancer samples [31].

By re-analysis of a large gene array dataset on human pancreatic cancer samples from van den Broeck et al. [24] a trend towards higher expression of IFIT3 in primary tumors of patients with poor outcome and in metastases was observed as compared to expression profiles in tumors of patients with fairly good survival. Although not statistically significant, this data strongly suggests that further investigation of the potential role of IFIT3 in pancreatic cancer is warranted.

Among the cytoplasmatic proteins shown to bind to IFIT3 are proteins capable of binding RNA, or involved in mRNA translation [21]. Here we provide data that IFIT3 also represents a candidate tumor promoting gene. Orthotopic injection of IFIT3 overexpressing pancreatic cancer cells led to the formation of tumors with greater mass and higher rates of metastasization (figure 3).

Transgenic overexpression of IFIT3 in COLO357FG cells rescued the cells from starvation-induced apoptosis and increased the proportion of mitotic cells (figure 1B). Knockdown of IFIT3 has been previously shown to moderate the expression of the apoptotic regulators caspases 3, 8, 9, and Bcl-2-associated X protein (BAX) [32]. Furthermore, IFIT3 expression is needed to maintain cell survival in Dengue virus (DV) infected cells. IFIT3 / IFIT2 heteromers negatively regulate the pro-apoptotic effects mediated by IFIT2 [33].

IFIT3 upregulation led to elevated secretion of VEGF (figure 1C). Tumor vascularization is needed to promote growth and expand tumor mass [1] and high levels of VEGF correlate with poor prognosis in pancreatic cancer patients [34].

The higher rates of liver- and lymph node metastases observed here by IFIT3 overexpression can partially be explained by both the anti-apoptotic effects of IFIT3, and augmented VEGF secretion. Resistance to apoptosis is a mandatory attribute that cancer cells need to acquire in order to survive the selection processes of the multi-step metastasization cascade [2]. VEGF expression on the other hand is known to be an important factor augmenting metastasization in pancreatic cancer [35].

Resistance to chemotherapeutic agents represents another trait of highly aggressive tumors. Gain of IFIT3 expression in COLO357FG cells yielded an increased resistance to chemotherapy. The exact mechanism by which this is mediated remains to be elucidated but the anti-apoptotic effects of IFIT3 by repression of pro-apoptotic regulators may provide an explanation [32]. STAT3-mediated expression of multidrug transporter MDR1 has been shown to facilitate resistance to chemotherapy in cancer cells [36]. Overexpression of IFIT3 led to elevated secretion of Interleukin-6, which is also mediated by STAT3 and therefore elevated MDR1 expression could be a possible explanation for chemoresistance observed.

Interestingly, a more profound desmoplastic reaction was seen in tumors derived from cancer cells with higher levels of IFIT3 expression. This points towards the promotion of a pro-inflammatory micromilieu within tumors by IFIT3 overexpression. This was supported by the observation that IFIT3 overexpression increases secretion of IL-6, which in turn favors pancreatic tumor growth and the maintenance of an inflammatory stimulus in the stromal tissue [37]. Elevated serum levels of IL-6 are correlated with poor prognosis in pancreatic cancer patients [38]. IL-6 drives inflammation in tumors, and enhances expression of VEGF, which further aggravates the disease [39].

STAT1, an important transcription factor for many interferon-stimulated genes, enhances IFIT3 expression by increasing the effects of the IRF-9/STAT2 complex and IRF-1 on the IFN-stimulated response elements (ISRE) [40, 41]. The NFκB protein c-Rel acts as a regulator for expression of several ISGs, including IFIT3 [28]. The expression levels of IFIT3 in pancreatic cancer cells were alterable as expected by IFN treatment, and by inhibition of STAT1 and NFκB signaling. This was also seen in L3.6pl, in which IFIT3 is constitutively expressed. This may be explained in part by the direct or indirect protein-protein interactions between IFIT3 and STAT1 as well as JNK documented here. This phenomenon points towards a self-regulatory process of IFIT3 in L3.6pl involving modulation of transcription controlled by STAT1, a known modulator of IFIT3 expression, and JNK/c-Jun, previously not known to be involved in IFIT3 regulation.

In search for an additional possible mechanism by which constitutive overexpression of IFIT3 is mediated in L3.6pl, our results demonstrate a key role of the transcription factor SOX9 in this process (figure 5). SOX9 was identified as a transcription factor that regulates hypoxia-related, pancreatic cancer-aggravating genes independently of hypoxia. This is thought to explain aspects of metastatic phenotypes [29]. Binding of SOX9 on the IFIT3 promoter was confirmed and silencing of SOX9 in L3.6pl had profoundly negative effects on the transcription levels of IFIT3 and diminished IFN induced IL-6 expression.

## CONCLUSION

IFIT3, which is known to be upregulated during inflammation and cellular stress of different kinds, appears to play an important role in pancreatic cancer biology. It acts as a potentially tumor promoting gene by mediating several malignant properties upon overexpression. Despite still being alterable by “classical” IFN or NFκB signaling, our findings indicate upregulation of IFIT3 without an adequate inflammatory stimulus in the more aggressive pancreatic cancer cell line. The transcription factor SOX9 is a key mediator of this “pseudoinflammatory” cellular condition that augments the extent of malignant behaviour of cells.

## MATERIALS AND METHODS

### Native and transfected cell lines

The human pancreatic adenocarcinoma cell lines COLO357FG and L3.6pl were cultured in DMEM under culture conditions and with supplements as previously described [9]. Cells were controlled for absence of Mycoplasma infection once per month by PCR.

IFIT3 overexpressing cell lines of COLO357FG and L3.6pl cells were produced using the CMV promoter containing pcDNA3.2 expression vector (Invitrogen, USA) in which the IFIT3 gene was cloned using the primer sequences described for PCR. Selection of the resultant transfected cell lines termed COLO357FG/CMV-IFIT3 and L3.6pl/CMV-IFIT3 was achieved by stepwise addition of G418 (Geneticin, Invitrogen, USA) until concentration reached 500 μg/ml. IFIT3 overexpression following transfection was confirmed by PCR and Western blot.

Regulation of SOX9 expression in L3.6pl pancreatic cancer cells was achieved using RNA interference as described [29]. Briefly, L3.6pl cells were stably transfected with the plasmid pcDNA6TR (Invitrogen, USA), then stably transfected with SOX9shRNA or LacZshRNA constructs and selected via Western blot (MoBiTech, Göttingen, Germany). Plasmid pENTRSOX9shRNA was created by ligation of oligonucleotides specific for the human SOX9 gene:

SOX9shRNAforw: CACCGCAAGCTCTGGAGACTTCTGACG AATCAGAAGTCTCCAGAGCTTG and

SOX9shRNArev: AAAAGCAAGCTCTGGAGACTTCTGATTCGT CAGAAGTCTCCAGAGCTTGC. The control plasmid pENTRLacZshRNA was prepared by insertion of oligonucleotides specific for *E. coli LacZ gene*:

LacZshRNAforw: CACCGGTTGTTACTCGCTCACATTTCGAAAA ATGTGAGCGAGTAACAACC and

LacZshRNArev: AAAAGGTTGTTACTCGCTCACATTTTTCG AAATGTGAGCGAGTAACAACC.

The culture medium of transfected cell lines termed L3.6pl-6TR-SOX9shRNA and L3.6pl-6TR-LacZshRNA contained 10 μg/ ml Blasticidin and 100 μg/ ml Zeocin for selection. Expression of SOX9shRNA or LacZshRNA was induced by adding 10 μg/ml of tetracycline to cell culture for 48 hours. Inhibition of SOX9 expression under this treatment was confirmed by Western blot.

### RNA extraction and PCRs

RNA was isolated from COLO357FG and L3.6pl cells using the RNeasy kit (Qiagen, Hilden, Germany) according to the manufacturers instructions. RNA integrity was verified by agarose-formaldehyde electrophoresis detection of 18S and 28S rRNA, and isolated RNA was quantified by spectrophotometry using the GeneQuant Pro RNA/DNA calculator (Pharmacia, Freiburg, Germany).

Semi-quantitative PCR was performed using the SuperScript® III One-Step RT-PCR System with Platinum® Taq High Fidelity Kit (Invitrogen, USA) according to the manufacturers instructions.

Quantitative RT-PCR was performed using the SuperScriptIII Platinum SYBR green one-step qR-PCR kit (Invitrogen, USA) with gene specific primers according to the manufacturer's instructions on a LightCycler system (Roche Diagnostics, Mannheim, Germany). Appropriate results were obtained after 45 cycles. PCR efficiency was assessed using the plasmid standards and quantified in relation to the house keeping gene transcript GAPDH. To check the size and identity of the PCR product, semi-quantitative RT-PCR was performed with the SuperScriptIII One-Step RT-PCR System with Platinum Taq High Fidelity (Invitrogen, USA) using the same template and primer sets mentioned above. All the PCR products were sequenced.

Primer sequences used for PCRs:

IFIT3 forward GAAGGAACTGGGCCGCCTGCTAAG

IFIT3 reverse GCCCTGGCCCATTTCCTCACTACC

GAPDH forward GAGTCAACGGATTTGGTCGTATTGGGCG

GAPDH reverse GACGCCTGCTTCACCACCTTCTTGATGTC.

Β-actin forward: GATGATGATATCGCCGCGCTCGTCGTC

Β-actin reverse: CTCGCGGTTGGCCTTGGGGTTCAG

SOX9 forw: CCGGGCCCGCGTATGAATCTCC

SOX9 rev CCGCCTGCGCCCACACCAT

### Re-analysis of gene array data from human pancreatic cancer samples for IFIT3 expression

The published data sets from van den Broeck et al. (GEO accession number 42952) were re-analyzed for expression profiles of IFIT3 [24]. The datasets contain raw data from gene arrays performed on whole pancreatic cancer samples stratified by good and poor clinical outcome as well as gene arrays performed on liver- and peritoneal metastases. From these datasets we re-analyzed the expression profiles of IFIT3 as well as UBB, CDH4, GAPDH, and PFDN1 as housekeeping genes as control. Quality control and clustering analyses were carried out using the MADMAX (Management and Analysis Database for Multi-platform microArray eXperiments) platform (https://madmax.bioinformatics.nl, University of Wageningen). The same platform was used to normalize the data using the RMA algorithm [42].

### IFIT3 expression

Cell lines were cultivated until confluence reached ~50%. Cells were subjected to growth factor starvation with only 0.5% FCS for 24 hours. Expression of *IFIT3* gene was induced by treatment with 0,2 IU/ml interferon α2a (Sigma-Aldrich, USA) for 24 hours [25]. Activity of the transcription factor NFκB was inhibited by treatment with 5μM BAY11-7082 for 48 hours (Enzo Life Sciences, Lörrach, Germany) [43]. Cytokine-induced activation of STAT1 was inhibited using 20μg/ml S14-95 for 48 hours [44] (Enzo Life Sciences, Lörrach, Germany). Expression data were compared with cells treated with vehicle as negative control.

### Immunoblotting

For Western blotting analysis proteins from lysated L3.6pl pancreatic cancer cells were separated in SDS-PAGE gel with an appropriate concentration of Acrylamide/BIS to ensure optimal separation. Proteins were then transferred onto PVDF membrane Hybond P (Amersham GE, Uppsala, Sweden) via semi-dry blotting. Efficient blotting was demonstrated by transfer of the PeqGOLD pre-stained protein marker IV (PeqLab, Erlangen Germany). Transferred proteins were subjected to immunodetection using WesternDot^TM^ 625 goat anti-mouse western blot kit (Invitrogen, USA). Briefly, membranes were blocked, then incubated for 1 hour with mouse anti-human IFIT3 antibody (Sigma-Aldrich, USA, Cat. No. SAB1405993-50UG), extensively washed and incubated for 1 hour with biotinylated goat-anti mouse antibody, washed and incubated for 1 hour with Qdot625 streptavidin-conjugate. After the last washing results were visualized using an UV-transiluminator (Bachofer, Germany).

Co-immunoprecipitation was performed using the Pierce® Co-Immunoprecipitation Kit (Thermo Scientific, USA), which helps to eliminate antibody contamination from IP-experiments due to the covalent binding of capture antibodies directly to the beads, with anti-human IFIT3 antibody (Abcam, UK, Cat. No: ab76818) with the help of the manufacturer's protocol to isolate IFIT3-protein complexes from the lysate of L3.6pl pancreatic cancer cells. After protein separation in SDS-PAGE, gel immunoblotting was performed as described using mouse anti-human STAT1 and mouse anti-human JNK antibodies (both from Cell signaling, USA).

### Protein affinity chromatography

Binding partners of the IFIT3 protein were identified using the One-STrEP set (IBA, USA) according to producer's instructions. Briefly: the human IFIT3 gene was inserted into the pEXPR-IBA103 vector at its BsaI site using in frame PCR cloning leading to a fused gene coding for the IFIT3 protein fused on its C-end with the One-STrEP Tag. Purification of the gene product was performed using the QIAquick PCR Purification Kit (Qiagen, Germany). The following primers were used in the process:

IFIT3IBAF: ATGGTAGGTCTCAAATGAGTGAGGTC ACCAAGAATTCCCT

IFIT3IBAR: ATGGTAGGTCTCAGCGCTGTTCAGTTGC TCTGAGTTAGAGAG

The resulting plasmid pEXPR-IFIT3 as well as the empty vector pEXPR-IBA103 were transfected to L3.6pl cells. Expression of the One-STrEP-tagged fusion protein was verified using western blotting and immunodetection with an anti-IFIT3 antibody and anti-STrEP antibody (IBA, USA). Cell lysates were prepared from both transfected cell lines, bound on affinity columns and extensively washed. The IFIT3-protein complexes were eluted using biotin buffer and analyzed by western blot analysis using mouse anti-human Akt, EGFR, Src, MAPK, STAT1 and JNK antibodies (all from Cell signaling, USA). Lysates from L3.6pl cells transfected with the empty vector were used as a negative control. Immunoprecipitation with anti-JNK and anti-STAT1 followed by Western blot using anti-IFIT3 was performed to confirm the results.

### FACS analysis for apoptosis, cell cycle and chemoresistance

For apoptosis and cell cycle analyses, native tumor cells were cultivated in 6-well plates with or without treatment with tetracycline, respectively. After washing and scraping into Nicoletti buffer, stained DNA content was analyzed by flow cytometry (FACSCalibur, BD Biosciences, USA). Portions of apoptotic (sub-diploid), G_0_/G_1_ phase (diploid), S phase (more than diploid) and M (tetraploid) phase cells were quantified using the WinDMI 2.8 software (Scripps Research Institute, La Jolla, USA).

For chemoresistance analyses COLO357-FG, FG-IFIT3 and L3.6pl at 70% confluence were treated with increasing concentrations of gemcitabine (Gemzar^©^, Lilly USA), 5-FU and irinotecan (both from Sigma-Aldrich, USA). The proportion of apoptotic cells was assessed using FACS analysis as described. The fold increase of apoptotic cells under increasing concentrations of chemotherapy compared to the amount of apoptotic cells with no treatment was calculated. One-way ANOVA was performed to analyze chemosensitivity curves for significant variances (GraphPad Prism 6.0, USA).

### VEGF, TNF-α and cytokine ELISA

Supernatants of cultured COLO357FG/CMV-IFIT3 and L3.6pl/CMV-IFIT3 as well as their empty vector controls were subjected to ELISA analysis for VEGF. To this end, the Quantikine human VEGF ELISA kit (BenderMedsystems, Vienna, Austria) was used according to the manufacturer's protocol. VEGF content was calculated according to the calibration curve. Calibration curves with a correlation coefficient of at least 0.998 were used.

Concentration of IL-1ß, IL-2, IL-6 and TNF-α was measured with and without addition of IFN-α2a at a concentration of 0,2 IU/ml as well as in L3.6pl-6TR-SOX9shRNA and L3.6pl-6TR-LacZshRNA with and without IFN-α2a and 10μg/ml tetracycline, respectively. Measurements were performed using the respective human cytokine platinum ELISA kits© (BenderMedsystems, Austria). Optical density was measured on an ELISA reader (Tecan, Crailsheim, Germany),

### Chromatin immunoprecipitation

The ChIP-IT Express enzymatic kit (Active Motif, USA) was used according to the producer's manual [45]. Briefly, L3.6pl cells were subjected to treatment with 37% formaldehyde containing fixation solution [46]. Cross-linked complexes of protein and DNA were isolated and sheared using the nuclease enzyme cocktail provided. Shearing was controlled by electrophoresis in 2,5% agarose gel after cross-link reversal. DNA-fragments cross-linked with bound proteins were purified via immunoprecipitation using anti-SOX9 antibody (Tebu-bio, Offenbach, Germany). Anti-tetracycline receptor antibody was used as an arbitrary control. Antibody-purified DNA-protein complexes were subjected to cross-linking reversal by treatment in high salt buffer by elevated temperature. Bound proteins, which could act as inhibitors of PCR were degraded via proteinase K treatment. DNA was purified and subjected to diagnostic PCR aiming to detect IFIT3-promoter. Used primers were:

IFIT3PF: GGGGCAGCTTCCAAGTCATAGG

IFIT3PR: GATTACAGGCACCCACCACCAT

PCR with no added template, unpurified chromatin after cross-linking reversal, control IgG added into immunoprecipitation reaction were run as controls.

### Orthotopic tumor model

The regional ethics authorities approved all animal procedures. To assess the influence of IFIT3 overexpression on orthotopic tumor growth, COLO357FG/CMV-null, COLO357FG/CMV-IFIT3 as well as L3.6pl/CMV-null were injected into the pancreas of male athymic BALB/c nu/nu mice (Charles River WIGA, Sulzfeld, Germany), as described previously [9]. Briefly, a left abdominal flank incision was made, the spleen was exteriorized, and 8 × 10^5^ tumor cells diluted to 40μl of PBS were injected into the subcapsular region of the pancreas. On day 37 after tumor cell injection, animals were sacrificed and examined for orthotopic tumor growth, lymph node (pyloric/pancreatic region, mesenteric, renal and lumbar nodes), and hepatic metastases.

### Statistics

All results are presented as mean values (±SD) unless otherwise indicated. Statistical significance was tested using student's t-test in cell culture experiments, one-way ANOVA with Tukey's HSD post-hoc test for multiple comparison in chemosensitivity assay, Mann-Whitney-U test to test for differences in tumor weight and Fisher's exact test for metastasization status. A two-tailed p-value<0.05 was considered significant (*p), p<0.001 highly significant (**p).
